# A bibliometric analysis of m6A methylation in viral infection from 2000 to 2022

**DOI:** 10.1186/s12985-024-02294-1

**Published:** 2024-01-18

**Authors:** Xing Tao, Gang Wang, Wudi Wei, Jinming Su, Xiu Chen, Minjuan Shi, Yinlu Liao, Tongxue Qin, Yuting Wu, Beibei Lu, Hao Liang, Li Ye, Junjun Jiang

**Affiliations:** 1 Guangxi Key Laboratory of AIDS Prevention and Treatment, School of Public Health, Guangxi Medical University, Nanning, Guangxi China; 2 China (Guangxi) - ASEAN Joint Laboratory of Emerging Infectious Diseases, Guangxi Medical University, Nanning, Guangxi China; 3 Biosafety Level -3 Laboratory, Life Sciences Institute, Guangxi Medical University, Nanning, Guangxi China

**Keywords:** N6-methyladenosine, Methylation, Viral infection, Bibliometric analysis, Data visualization

## Abstract

**Background:**

N6-methyladenosine (m6A) methylation has become an active research area in viral infection, while little bibliometric analysis has been performed. In this study, we aim to visualize hotspots and trends using bibliometric analysis to provide a comprehensive and objective overview of the current research dynamics in this field.

**Methods:**

The data related to m6A methylation in viral infection were obtained through the Web of Science Core Collection form 2000 to 2022. To reduce bias, the literature search was conducted on December 1, 2022. Bibliometric and visual analyzes were performed using CiteSpace and Bibliometrix package. After screening, 319 qualified records were retrieved.

**Results:**

These publications mainly came from 28 countries led by China and the United States (the US), with the US ranking highest in terms of total link strength.The most common keywords were m6A, COVID-19, epitranscriptomics, METTL3, hepatitis B virus, innate immunity and human immunodeficiency virus 1. The thematic map showed that METTL3, plant viruses, cancer progression and type I interferon (IFN-I) reflected a good development trend and might become a research hotspot in the future, while post-transcriptional modification, as an emerging or declining theme, might not develop well.

**Conclusions:**

In conclusion, m6A methylation in viral infection is an increasingly important topic in articles. METTL3, plant viruses, cancer progression and IFN-I may still be research hotspots and trends in the future.

**Supplementary Information:**

The online version contains supplementary material available at 10.1186/s12985-024-02294-1.

## Introduction

Viral infection refers to the process in which viruses invade the body through various channels and proliferate in susceptible host cells [1]. This can occur at the site of entry, also known as a localized infection, or the virus can spread throughout the body, causing a systemic infection. Viral infections commonly cause respiratory and digestive illnesses, but viruses can also infect most other parts of body [2]. United Nations scientists have warned that there may be 1.7 million undiscovered viruses in nature, half of which could infect humans and spark new epidemics [3, 4]. However, the pathogenesis of many viral diseases caused by viral infections remain unclear, and it is urgently essential to keep up with the current hotspots to explore the pathogenesis and complications of viral diseases [5].

N6-methyladenosine (m6A) is the most common and reversible internal modification of mammalian messenger and noncoding RNAs. m6A RNA modification regulates RNA splicing, translocation, stability and protein translation [6, 7]. The N6-methylation of adenosine is catalyzed by a 200 kDa methyltransferase heterodimer complex consisting of the methyltransferase-like protein 3 (METTL3), methyltransferase-like protein 14 (METTL14), wilms tumor 1 associated protein, RBM15, ZC3H13, KIAA1429, and METTL16 [8–10]. The removal of m6A is catalyzed by two demethylases, α-ketoglutarate-dependent dioxygenase homolog 5 and the fat mass and obesity-associated protein (FTO) [11, 12]. m6A modification in mRNA can be specifically recognized by HNRNPC/HNRNPA2B1, members of the heterogeneous nuclear ribonucleoprotein family of proteins and IGF2BP1/2/3, members of the YT521-B homology (YTH) family of proteins [13]. Many human diseases are associated with altered m6A modification, and it provides a potential new pathogenesis research for the prevention or treatment of various diseases, such as cancer, cardiovascular disease, autoimmune diseases, metabolic diseases, and infectious diseases [14–17].

As viral infection represents a significant global health burden, its complex pathogenesis and the long-lasting multi-organ damage caused by it have made us pay more attention to the driving mechanism of viral infection. As the most common methylation modification in eukaryotes, and also a new therapeutic target in recent years, made m6A an increasingly interesting topic with a measurable increase in publications. Investigations now encompass viruses from retroviruses, DNA viruses, positive-sense RNA viruses, to negative-sense RNA viruses across various viral families. Enabled by advancements such as PA-m6A-seq, miCLIP, m6A-LAIC-seq, and SELECT, comprehensive transcriptome-wide mapping of m6A modification sites in various viral types has emerged. Leveraging mutations to eliminate m6A modification sites within viruses, scientists have uncovered distinct roles played by m6A modifications across diverse viral species. Notably, m6A modifications exert positive regulatory effects on viruses like vacuolating virus 40(SV40) [18], enterovirus 71 (EV71) [19], influenza A virus(IAV) [20], Rous sarcoma virus(RSV) [21], and human metapneumovirus (HMPV) [22], while exhibiting negative regulatory impacts on viruses such as hepatitis C virus (HCV) [23] and Zika virus (ZIKV) [24]. Differential impacts of m6A modifications within the same virus across distinct life cycles or varied culture systems have been observed. For instance, within hepatitis B virus (HBV), m6A modifications in the 3’UTR region diminish RNA stability, while exhibiting a positive regulatory role in pgRNA reverse transcription within the 5' epsilon loop [25]. Similar regulatory mechanisms have been noted in viruses like human immunodeficiency virus 1(HIV-1) [26, 27] and Kaposi’s sarcoma-associated herpesvirus (KSHV) [28, 29]. Accumulating evidence from these studies indicates that m6A modifications can influence viral genome replication and transcription, modulate the host cell’s immune response, and regulate the physiological state of host cells post-viral infection, exacerbating the viral infection’s impact on the host. Hence, an urgent need exists to comprehensively review and summarize the m6A methylation modifications in viral infections based on existing literature, providing a systematic overview and synthesis of key findings.

Different from traditional systematic reviews, the bibliometric analysis comprehensively quantifies and discovers research hotspots and trends in the target field through mathematical techniques [30]. It not only helps researchers to grasp the hot spots and trends in a specific research field, but also helps to reveal the quantitative relationship, distribution structure and changing rules of articles and information [31, 32].This method of analysis has been widely used in various fields to develop guidelines, identify research hotspots and assess research trends [33]. Therefore, this study took the articles related to the role of m6A methylation in virus infection as the research object, and used the bibliometric analysis method to analyze the characteristics of publication output. The hotspots and development trends of research topics in this field were clarified through the analysis of author’s keywords and keyword plus, so as to determine the research focus. This study identifies important evidence for m6A methylation in viral infection, helping scholars understand the intellectual background and emerging research trends in this field.

## Methods

### Data sources

The data were obtained from the Web of Science Core Collection (WoSCC) in this study. Due to the rapid update of this database, the literature search was performed on 1 December 2022 to avoid bias. The search terms were as follows: [ALL = (‘m6A’ OR ‘m(6)A’ OR ‘N-6-methyladenosine’ OR ‘N6 -methylation’ OR ‘N-6 methylation’) AND ALL = (‘viral infection’ OR ‘virus’ OR ‘viruses’)]. Only original articles and reviews in English were included.

### Data collection

In this study, the comprehensive publishing characteristics were extracted from the WoSCC database, including the number of articles and citations, H-index, year of publication, country, affiliation, author, journal, references, and keywords. Search results for the target subject were recorded in ‘Full Record and Cited References’, while files were saved and renamed in ‘Plain Text’ file format. The initial data preprocessing involved filtering articles based on the specified criteria. Articles of the types ‘article’ and ‘review article’ were selected, and the language parameter was set to ‘English’ to restrict the downloaded literature to these categories. Irrelevant articles not associated with m6A methylation and viral infections were subsequently removed. The processed data was then imported into CiteSpace software, with the previously established ‘input’ and ‘output’ folders placed in their respective locations within the software. To ensure data integrity, duplicates were removed, addressing redundancies across articles, reviews, and conference papers. The cleaned dataset was then exported for further analysis. CiteSpace software and the bibliometric analysis capabilities of the R software package were employed for literature metrics, visual analysis, and content analysis.

### Bibliometric analysis

Bibliometric and visual analyzes were performed using CiteSpace software (version 6.1.R3) and the R-based Bibliometrix (4.2.1) package. As a bibliometrics analysis software, CiteSpace can analyze the knowledge database, research content, hotspots and frontiers of a specific research field through a visualized network map. In this study, CiteSpace was used to obtained dual-map overlay of citations and visualize keywords, clusters, and partnerships between institutions and countries. Subsequently, the Bibliometrix R package was used to conduct author's keywords analysis, theme evolution, multiple correspondence analysis, and thematic map to obtain trending topics and future development directions in related research fields. GraphPad Prism 9.0 was used to visualize the annual publication output. JMP 14 Pro software was used to draw the gengrahical distribution of article publications.

## Results

### The trend of publication outputs

**Fig. 1:**
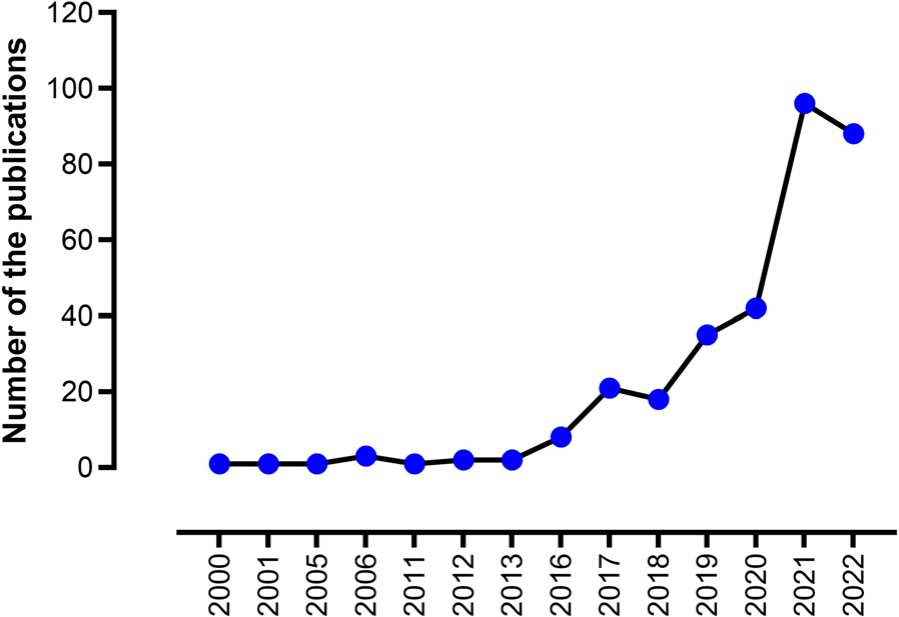
The trend of annual publications related to m6A in viral infection

A total of 320 documents were collected from the Web of Science Core Collection based on the predefined queries. Excluding 1 duplicate record, 319 eligible records were included in this review. As shown in Fig. 1, the research trend was divided into three stages. The first stage with only 11 researches was from 2000 to 2013, when the average output of documents was 1.6 per year. In the second stage, the literature output increased slightly. There were 8 articles in 2016, 21 articles in 2017, and a drop of 18 articles in 2018, and then increased to 35 and 42 articles in 2019 and 2020, respectively. After 2020 was the third stage, in which 96 articles were produced in 2021, which was twice the output in 2020. Although the output in 2022 was slightly lower than that in 2021, the total output was still large, which indicates that m6A is a promising direction in the field of viral infection.

### Country/regional distribution analysis of publication outputs

**Fig. 2:**
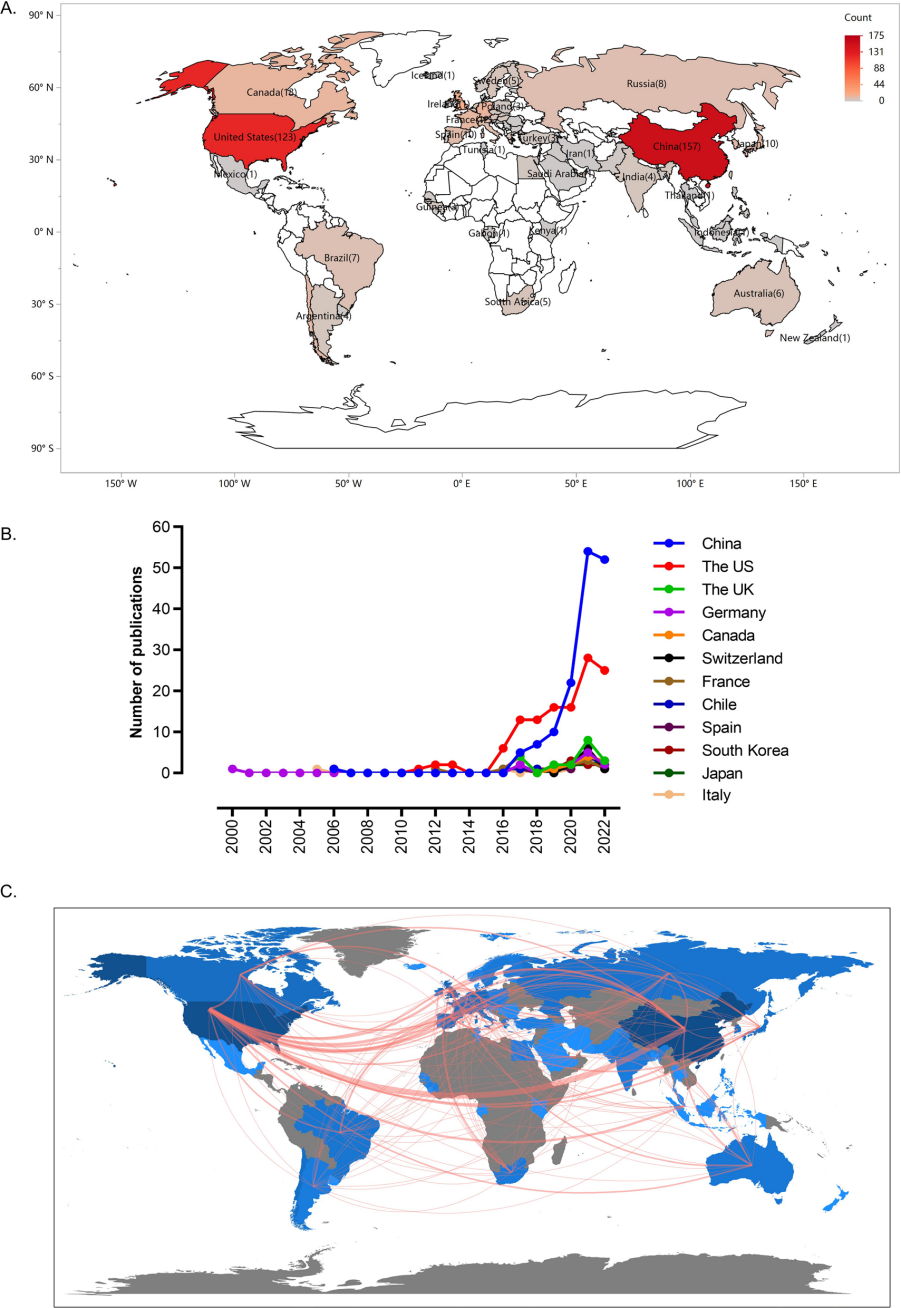
Geographical distribution of article publications related to m6A in viral infection and the map of collaboration across countries. **A** Geographical distribution of article publications. The label infers to the country and the number of articles published by this country, and the depth of color matches the volume of the publications. **B** The trend of annual publications of the top 10 countries related to m6A in viral infection. The Spain, South Korea and Japan contribute the same volume of the publications. **C** Visualization map of collaboration across countries. Red links represent partnerships between the two regions. The depth of color matches the number of published articles, gray areas indicate regions with no output

By categorizing literature based on its publication location and subsequently calculating metrics such as the number of publications, proportion, and growth rate for each category, we aim to gain insights into the research output of different countries or regions in a specific academic discipline or field. 319 records were published in 28 countries. The darker color in Fig. 2A indicates that the country has higher publications, while white indicates no publication output. China ranked first with the highest number of 157 articles in this field. The US with 123 articles ranked second, and The UK ranked third with 19 articles. China and the US were far ahead of other countries. The trend of annual publications of the top 10 countries was shown in Fig. 2B. Germany was the first country to publish articles, but the number of papers published globally did not show an upward trend until 2016. As of 2019, the annual output of the US was significantly higher than any other country, and then China overtook other countries to ranked first in annual publication outputs.

Subsequently, a country-by-country collaboration analysis was performed (Fig. 2C). Among all countries, the US had the greatest centrality (0.44), followed by Israel (0.43) and China (0.28), indicating that they have a strong bridge role in this area. It is worth mentioning that despite relatively few published articles, Israel had close cooperation with other countries, especially with regions such as Cyprus, Croatia, Bangladesh and Bulgaria. In this field of research, the US had a higher central status and more cooperation, such as China, Germany, Canada, France and Switzerland.

## Keyword co-occurrence and analysis

Keyword co-occurrence analysis involves conducting a statistical examination of keywords across multiple documents to understand the relationships between different terms, thus reflecting the knowledge structure and developmental trends within a specific academic discipline or field. This analysis illuminates the degree of association among diverse keywords, enabling the identification of research focal points, emerging study areas, and the evolving trends in the knowledge structure of the discipline or field. Keywords represent the hot topics and fields of research. We used CiteSpace to extract and count the keywords in the articles, and merged the top similar keywords to get the top 20 high-frequency keywords as shown in Additional file 1: Table S1. ‘Nuclear RNA’ ‘methylation’ ‘gene’ ‘messenger RNA’ and ‘viral replication’ had higher frequency. Table 1 showed the top 20 keywords with the strongest citation bursts, which reflect and predict the staged hotspots and evolution trends of m6A methylation in viral infection. ‘Sequence specificity’ ‘infection’ and ‘methylation’ with stronger bursts of strength emerged earlier and were the topics of early attention. The keyword ‘sequence specificity’ had the longest duration of 13 years. Since 2020, ‘innate immune’ ‘DNA’ and ‘mettl3’ have appeared in the keywords, and the strongest burst (Strength = 3.54) indicated that it was the hotspot and maybe a turning point with prospective research implications.

**Table 1:**
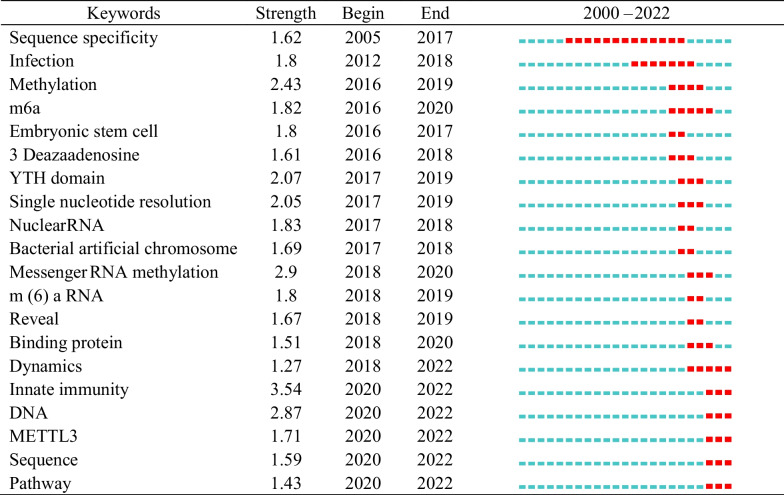
Top 20 keywords with the strongest citation bursts.

**Fig. 3:**
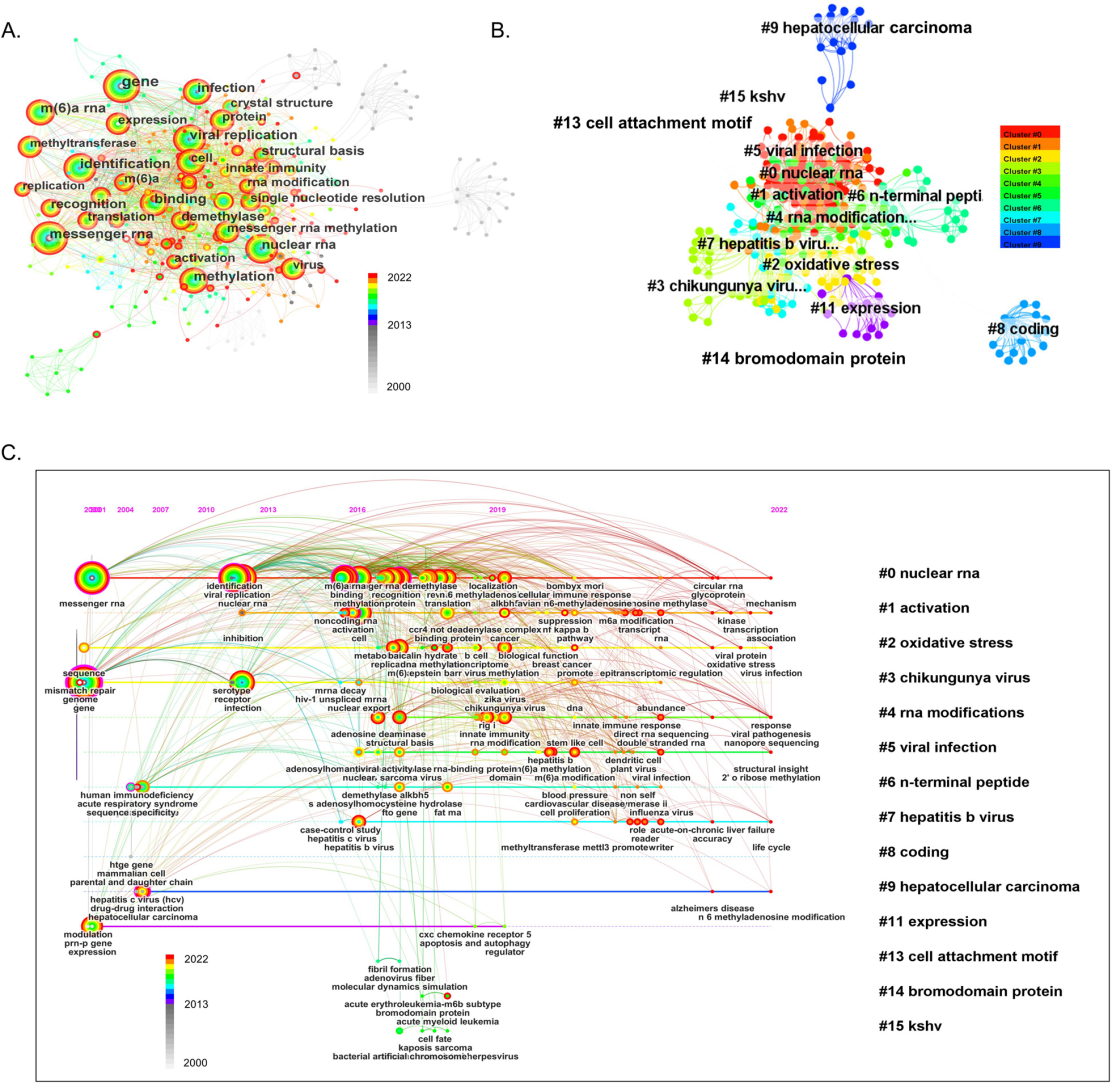
Visualization maps of keyword co-occurrence and cluster analysis networks. **A** Keyword co-occurrence analysis. Each node with colorful annual rings represents a keyword. The size of the nodes matches the publications outputs. The separate areas made up of nodes and links represent the relationship of different keyword. **B** Keyword cluster analysis. All keywords are divided into 15 clusters. The nodes and edges of different colors represent different clusters. **C** Visualization timeline view of keywords clustering analysis related to m6A methylation in viral infection. Horizontal lines of different colors with labels represent clusters formed by keywords, nodes on the horizontal line represent keywords, and the positions of nodes on the horizontal line represent the year when documents containing keywords first appeared, thus forming the timeline of the keyword clusters evolution

Keyword co-occurrence analysis helps researchers identify central issues and developments in a subject. The map with a network density of 0.0221 comprised 407 nodes and 1828 links (Fig. 3A). To explore the research theme, log-likelihood tests (LLR) were used to cluster the keywords of the published articles by CiteSpace software, and the clusters with different colors were shown in Fig. 3B. Cluster #0 nuclear RNA was the largest cluster, followed by cluster #1 activation and cluster #3 oxidative stress. Subsequently, the clusters were presented in a timeline view to observe the underlying knowledge structure and evolution over time of m6A methylation in viral infection (Fig. 3C). The nodes are chronicled on the horizontal line, which evolved the historical outcomes of the cluster. Cluster #0 nuclear RNA and #3 chikungunya virus appeared earliest and #4 RNA modifications appeared latest. Cluster ‘nuclear RNA’ ‘activation’ ‘oxidative stress’ ‘RNA modifications’ ‘viral infection’ ‘hepatitis b virus’ and ‘hepatocellular carcinoma’ related studies were available in 2022, while cluster ‘chikungunya virus’ ‘n-terminal peptide’ ‘coding’ ‘expression’ ‘cell attachment motif’ ‘bromodomain protein’ and ‘kaposi's sarcoma-associated herpesvirus (kshv)’ gradually decreased or even disappeared, suggesting a decreased trend in these fields. It was observed that the nodes of ‘messenger RNA’ ‘viral replication’ ‘sequence’ and ‘mismatch repair’ were larger, which indicated that there were more published articles on related studies. The number of nodes in cluster ‘viral protein’ ‘viral pathogenesis’ ‘innate immune response’ and ‘mechanism’ grew in recent years and may be the frontier on m6A methylation in viral infection in the future.

**Fig. 4:**
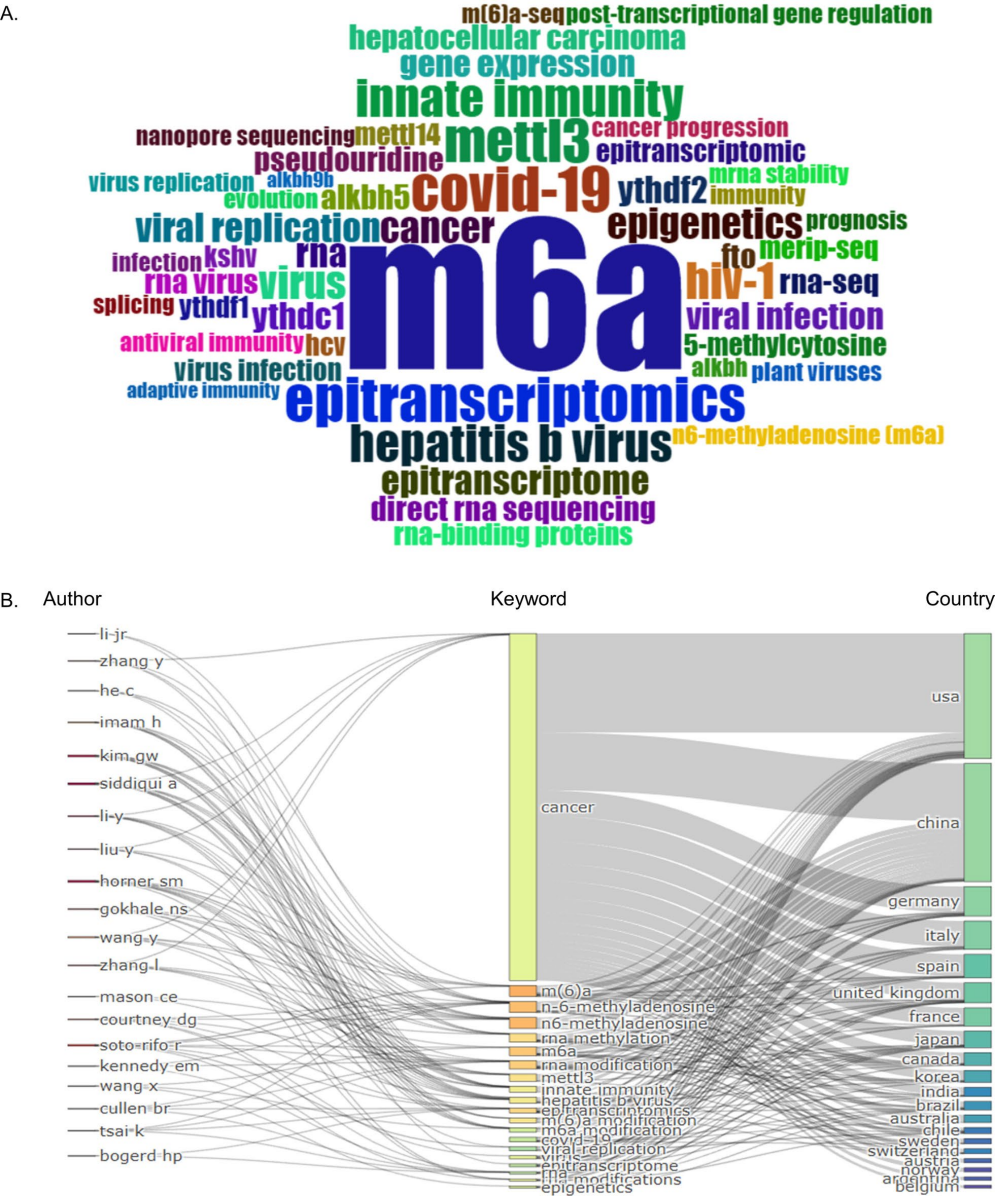
The word cloud and Sankey diagram of author’s keywords related to m6A in viral infection. **A** The author’s keywords of m6A in viral infection. Colors represent different keywords, and the size represents the frequency of keywords. The words with the highest frequency were m6A, followed by COVID-19, epitranscriptomics, METTL3, hepatitis B virus, innate immunity and HIV-1. **B** Sankey diagram of the association among keywords, authors and countries

Subsequently, the keywords with high frequency were counted using the bibliometrix R package. Different from the above analysis, the field selected in the specific R package is author’s keywords. The result showed that the keywords with the highest frequency were m6A (181), followed by COVID-19 (17), epitranscriptomics (17), METTL3 (15), hepatitis B virus (12), innate immunity (12) and human immunodeficiency virus 1 (HIV-1) (11) (Fig. 4A). Keywords, authors, and countries were then linked to a connected map, which enables discovery of which countries and authors are researching which aspect of m6A (Fig. 4B). The results could be used by policy makers to identify areas of research on this topic.

### Authors collaboration and cited authors analysis

**Fig. 5:**
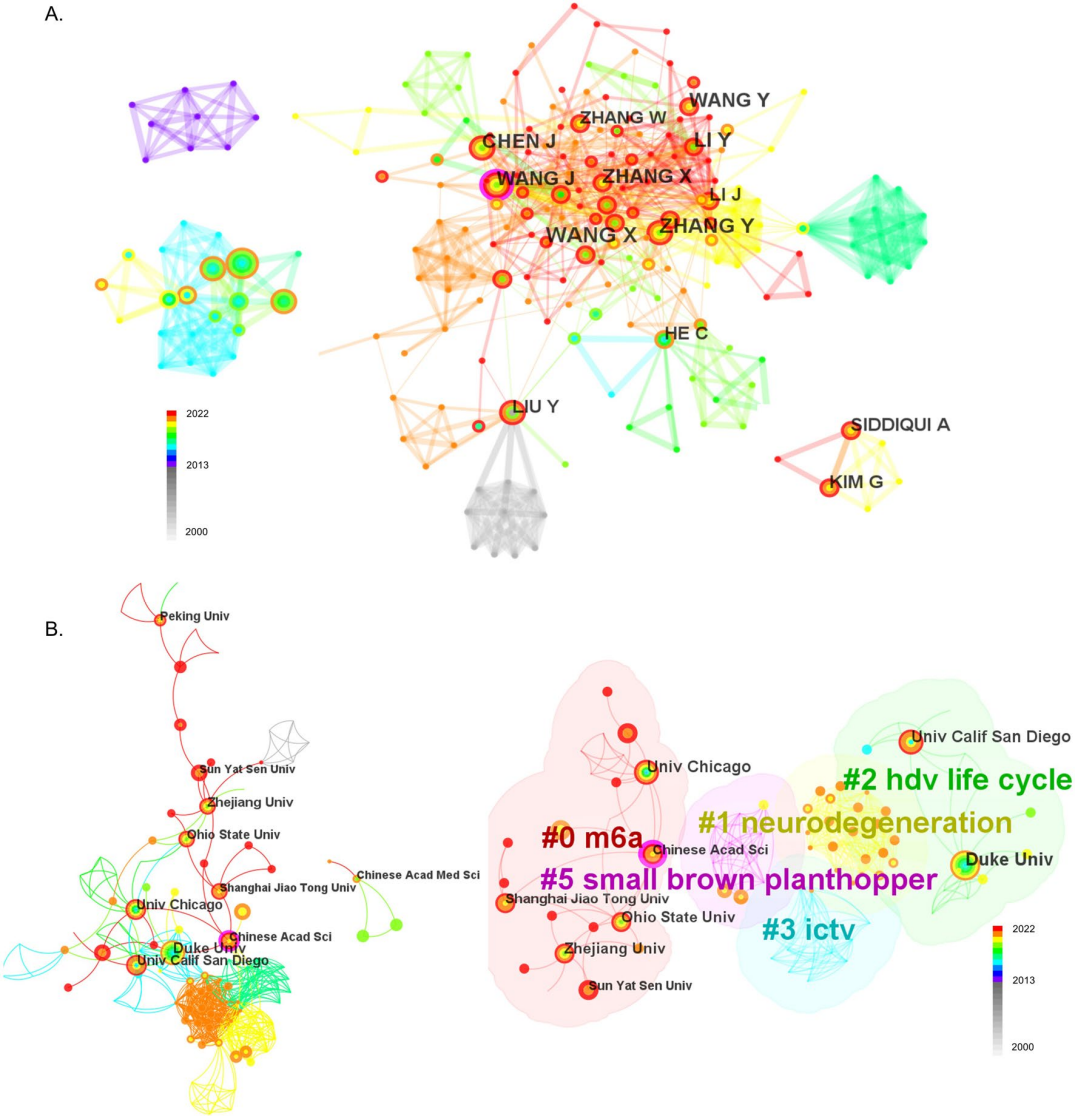
Authors collaboration analysis. **A** Visualization map of the top 7 author collaborations related to m6A methylation in viral infection. Each node with colorful annual rings represents an author. The size of the nodes matches the publications of the author. The nodes circled in purple represent greater centrality. The separate areas made up of nodes and links represent the author collaborative relationships. **B** Co-occurrence network analysis of institutions reveals collaborative relationships. The map, with a network density of 0.0232, consists of 236 nodes and 642 links. The largest node is the Chinese Academy of Sciences, circled in purple, with a centrality of 0.12, indicating that it plays a critical role in this field. Most institutions have collaborated, such as Duke University, Zhejiang University, University of California San Diego, and The University of Chicago (left). To explore the research topics between institutions, keywords were clustered using the log-likelihood ratio test (LLR), with ‘m6A’ as the keyword being the largest cluster (right)

Analysis of authors collaboration and cited analysis was used to measure the collaborative relationships among authors and the citation relationships among documents. This analysis serves to reflect the developmental trends, research focal points, and academic exchanges within a discipline by exploring the dynamics of collaboration and influence among researchers. These publications involved 328 authors, with an average of 2.65 articles per author. The first seven authors were all from China. Wang X was the highest producer with 17 papers. WANG X was the most cited author with 181 citations, followed by MEYER KD, DOMINISSINI D and LICHINCHI G (Additional file 2: Table S2). Then, the author collaborations were analyzed. Figure 5A showed the top 7 author collaboration groups. WANG X, ZHANG Y, and LI Y and others formed the largest collaborative team, of which WANG J was circled in purple for greater centrality. SIDDIQUI A, KIM D and others made up the second cooperative team, while HE C and LIU Y formed separate groups. Co-occurrence network analysis of institutions reveals collaborative relationships. The map, with a network density of 0.0232, consists of 236 nodes and 642 links (Fig. 5B). The largest node is the Chinese Academy of Sciences, circled in purple, with a centrality of 0.12, indicating that it plays a critical role in this field. Most institutions have collaborated, such as Duke University, Zhejiang University, University of California San Diego, and The University of Chicago (Fig. 5B left). To explore the research topics between institutions, keywords were clustered using the LLR, with ‘m6A’ as the keyword being the largest cluster (Fig. 5B right).

### Cited journals analysis

**Fig. 6:**
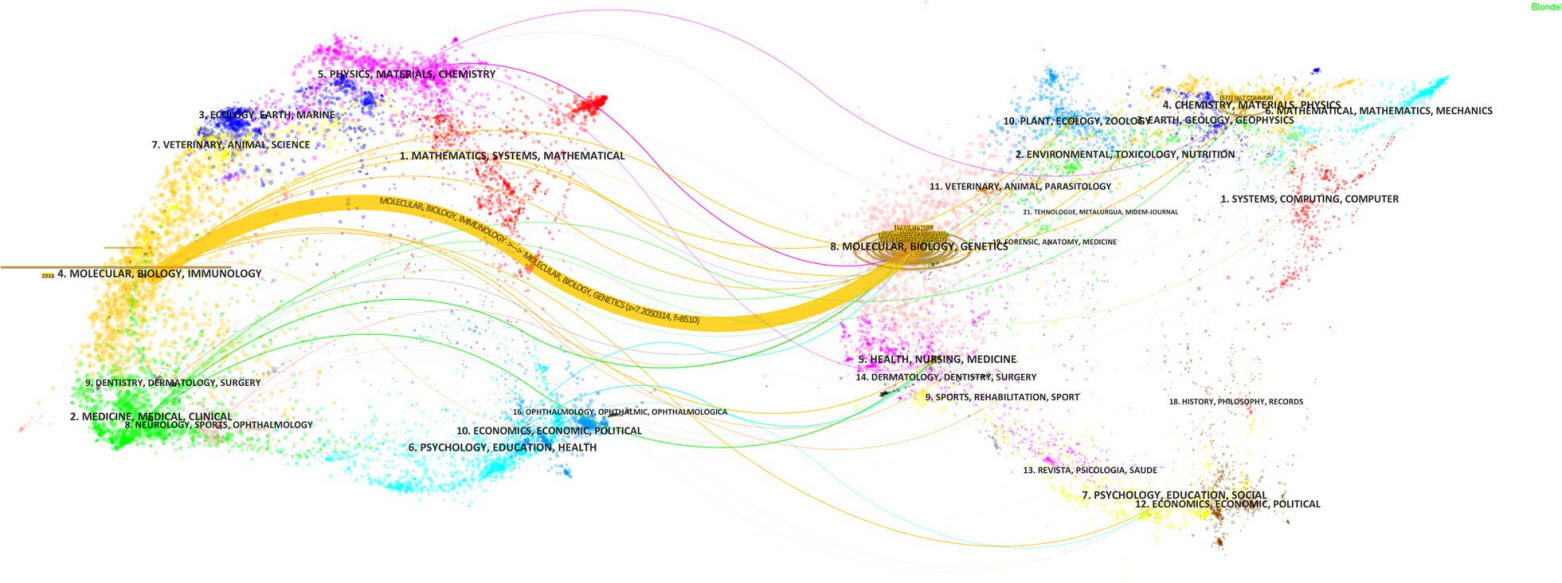
The dual-map overlay of journals related to m6A methylation in viral infection. Nodes on the left represent included documents, and nodes on the right represent references in the documents. Labels represent disciplines, and links represent the cited path

By analyzing cited journals, cited journal analysis can provide important reference information for subject development, research evaluation, academic exchanges, etc. Among the 576 cited journals, 26 were cited over 100 times (Additional file 3: Table S3). *Nature* was the most cited journal, followed by *Cell* and *Nucleic Acids Research*. Of the top 10 cited journals, 8 were in the Q1 region, while *Journal of Virology* and *Journal of Biological Chemistry* belonged to Q2. *Nature* had the highest impact factor (IF) of 69.504, and *Cell* ranked the second with an IF of 66.850. The dual-map overlay of journals reflects the relationship between the source journal (left) and the target journal (right), as well as the topics covered in the journals (Fig. 6). The colored links represent the relationship between the two journals, with 1 main broad citation path (yellow path). The result showed that journals involving molecular, biology and immunology were always cited by journals of molecular, biology and genetics, with a *Z*-value of 7.21 and an *f*-value of 8510. It is worth noting that we subsequently conducted an accessibility analysis to assess whether articles had been published in open-access journals, aiming to analyze the proportion of open-access publications in this particular research category. The results indicated that out of 319 articles, 245 (76.80%) were freely accessible in the database. This finding underscores the accessibility and impact of the literature in question, emphasizing the prevalence of open-access publications within the database.

### Theme evolution and thematic map

**Fig. 7:**
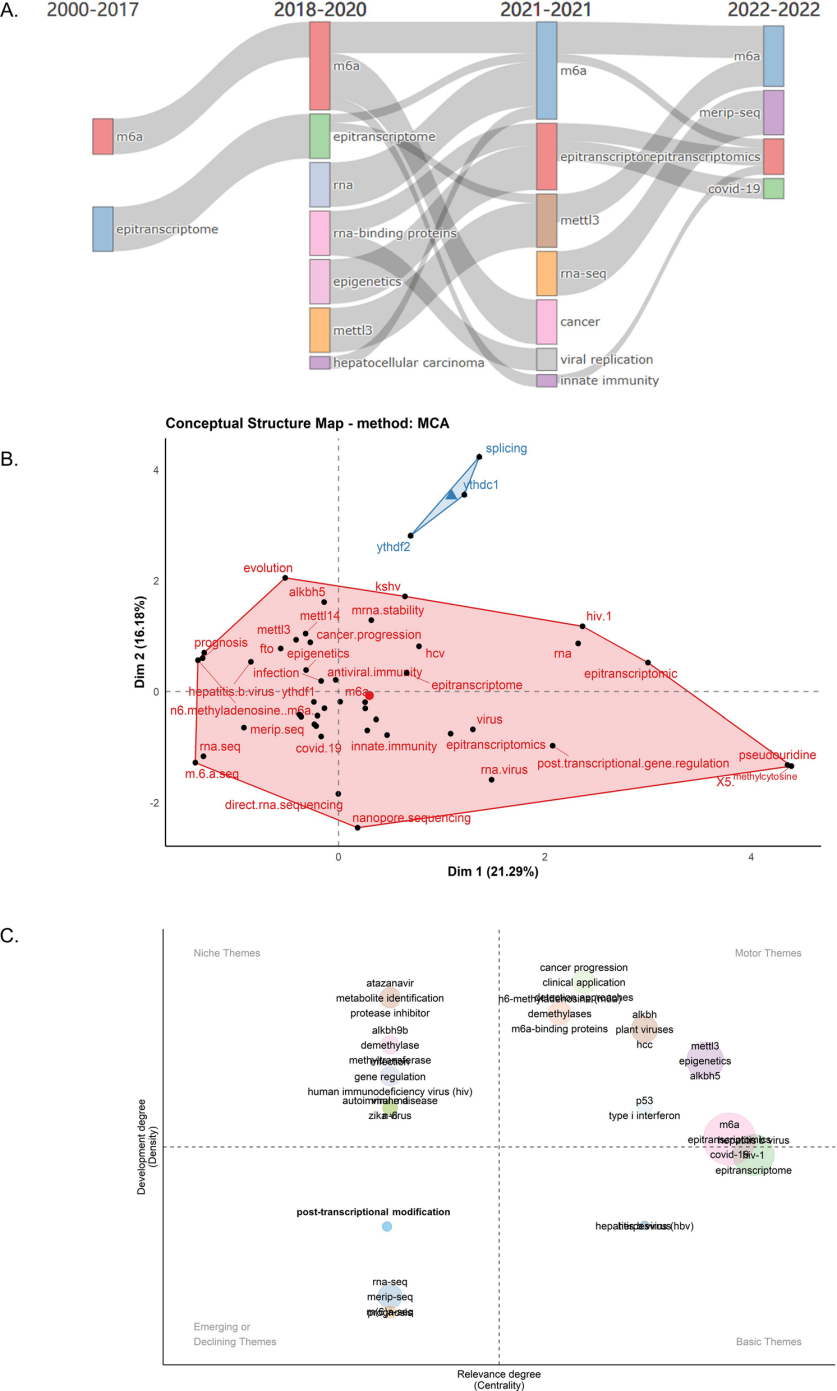
Theme evolution and thematic map. **A** Keyword evolution analysis of m6A methylation and viral infection in different time periods from 2000 to 2020. The nodes represent the main research topics generated from the co-occurrence network analysis, and the number of keywords contained in each node is represented by the size of the corresponding node. Time slices from adjacent segments sharing the same keyword are connected by streamlines, the width of which is proportional to the number of keywords. **B** Conceptual structure map. The area marked in red indicates the first largest category of keyword clusters, and the blue area indicates a small category. Each dot represents a keyword, and its distance means how often they appear in the article. The proximity of a keyword to the center point represents its popularity in the research field. **C** Thematic map. The horizontal axis represents centrality, and the vertical axis represents density. The first quadrant (upper right) is motor themes, implying both important and well developed. The second quadrant (upper left) is highly developed and isolated themes, indicating that there has been good development, but not important for the current research field. The third quadrant (lower left) is emerging or declining themes, indicating marginal themes that may not have a good development. The fourth quadrant (lower right) is basic and transversal themes, which are important to the field but have not been well developed (generally basic concepts)

As the discipline evolves, the usage of keywords or topics in the literature undergoes changes. Theme evolution and thematic maps can reflect the research interests and focal points within a disciplinary field. This provides valuable insights for the analysis of disciplinary development trends while uncovering research hotspots that guide future research directions within the field. The period from 2000 to 2022 was divided into 4 parts as shown in Fig. 7A. Before 2017, the keywords were m6A and epitranscriptome, and then the research on METTL3 and hepatocellular carcinoma related fields began to increase. In 2021, viral replication and innate immunity of viruses began to receive attention. METTL3 and epitranscriptome were still one of the research priorities. In 2022, the research on m6A methylation related to COVID-19 showed an increased trend and occupied an important position. Multiple correspondence analysis was performed to generate a conceptual structure map of author’s keywords (Fig. 7B). Inferred from the results, the keywords were divided into a large category and a small category. The first category marked in red involved most of the important concepts in the research field, which is highly consistent with the study of m6A methylation in viral infection, where m6A, innate immunity, METTL3/14, viral replication and hepatitis virus, etc. were important factors. In addition, prognosis, RNA binding proteins, post-transcriptional gene regulation, etc. have also attracted considerable research interest. Thematic map was implemented to assess the importance and future trends of keywords (Fig. 7C). The first quadrant, representing ‘motor themes’, signifies the research hotspots within the field, serving as focal points for future investigations. These areas exhibit high research density and significant influence, indicating substantial progress in the field and widespread academic attention. The second quadrant, labeled as ‘Niche themes’, indicates a solid research foundation within the field without clear dominance. While these areas demonstrate high research density, their impact is comparatively low, suggesting a cumulative body of research without breakthrough achievements. The third quadrant, denoted as ‘Emerging or declining themes’, reflects research in its early stages, requiring further development. These areas have low research density and low impact, signifying an exploratory phase that necessitates additional experience and accumulation of knowledge. The fourth quadrant, termed ‘Basic themes’, represents research with potential practical applications, warranting further exploration. Although these areas have low research density, their impact is high, indicating promising prospects but still in the nascent stages of development. In this study, the first quadrant (upper right) showed the most important themes, implying both important and well-developed. METTL3, plant viruses, cancer progression and type I interferon (IFN-I) were included, reflecting a good development trend and may be future research hotspots. The third quadrant (lower left) showed emerging or declining themes. post-transcriptational modification and RNA-seq might be defined as marginal topics.

## Discussion

The number of publications produced at a particular stage reflects the trends and interest in research topics. In this study, we found that the trend for annual publications is generally upward. The period from 2000 to 2016 was just a nascent period, and few articles were published. Then the number of published papers began to increase. After 2020, with the rapid growth of literature, the research on m6A methylation in viral infection has entered a critical period. It is clear that research related to m6A methylation in viral infection has been a hot topic in recent years and has a dynamic future trend. From the map analysis of country and institution distribution, we found that the researches of m6A methylation in viral infection have been conducted in many countries and regions. China contributed the most in this field with 157 publications, followed by the US with 123. The US ranks highest in terms of total link strength, suggesting that articles published in the US are likely to be more influential. Globally, more than 200 institutions study the role of m6A methylation in viral infection, among which Duke University has published the most, and Chinese Academy of Sciences has the highest centrality. It is likely that the contributions of scholars such as WANG X, ZHANG Y, LI Y, ZHANG X, WANG Y, WANG J, CHEN J, LI J, and SIDDIQUI A contributed to this result. Thematic map is used to assess future trend in this field.

Keywords clearly express the topic, hotspots and and frontiers of the document, and are also the most important section of bibliometrics. In the analysis of author’s keywords, m6A, COVID-19, epitranscriptomics, METTL3, hepatitis B virus, innate immunity and HIV-1 appeared frequently. Studies have shown that the SARS-CoV-2 virus has m6A modification enriched at the 3’ end of the viral genome. Loss of METTL3 in host cells reduced m6A levels in SARS-CoV-2 and host genes, and reduction of m6A in viral RNA increased RIG-I binding, which subsequently enhanced innate immune signaling pathways and inflammation-related gene expression, implying the association between m6A levels and the pathogenesis of COVID-19, which will help to achieve the intervention of COVID-19 from the perspective of innate immunity [22, 34]. Likewise, cells modulate host innate immunity to hepatitis B and C virus infection by inducing m6A modification of viral transcripts. Depletion of METTL3 and METTL14 leads to increased recognition of viral RNA by RIG-I, which stimulates the production of IFN-I, whereas readers YTHDF2 and YTHDF3 inhibit RIG-I recognition by occupying m6A-modified RNA [35]. In addition, m6A methylation has also received much attention in the regulation of innate immunity and antiviral responses, HBV life cycle, and hypoxia [25, 36–38]. In HIV-related studies, m6A modification of HIV RNA has been found to inhibit the expression of IFN-I in differentiated human monocytes and primary monocyte derived macrophages. The expression of IFN-I mRNA was significantly decreased in mononuclear cells transfected with a single m6A modified HIV RNA fragment. In addition, HIV with low m6A levels enhanced IFN-I expression, while HIV with high m6A levels had the opposite effect. m6A of HIV RNA escapes RIG-I-mediated RNA induction and activation of transcription factors IRF3 and IRF7 that drive IFN-I gene expression, suggesting the role of m6A modification of HIV RNA in evading innate immune responses in myeloid cells [39].

Thematic map was implemented to assess the importance and future trends of keywords. All topics are divided into four quadrants. The first quadrant (upper right) is motor themes, implying both important and well developed. The second quadrant (upper left) is highly developed and isolated themes, indicating that there has been good development, but not important for the current research field. The third quadrant (lower left) is emerging or declining themes, indicating marginal themes that may not have a good development. The fourth quadrant (lower right) is basic and transversal themes, which are essential to the field but have not been well developed (generally basic concepts). The result showed that topics such as METTL3, ALKBH5, hepatocellular carcinoma, plant viruses, cancer progression, clinical application, and IFN-1 reflect good development trends and may be future research hotspots. Whereas post-transcriptional modification is in the third quadrant, indicating borderline themes that may not develop well.

m6A is the most abundant RNA modification in eukaryotes, and many studies have shown that it has an essential impact on virus replication [40, 41]. About 40 years ago, m6A modification was found in viral RNA. In recent years, technological advances have made m6A a research focus to elucidate the role of this RNA modification in viral epitranscriptomics, such as IAV, HIV-1, HCV, ZIKV, etc. [20, 23, 24, 42]. IAV, as the first virus discovered to express mRNA with m6A, has been mapped to the m6A site in the mRNA and vRNA strands and demonstrated that m6A modification increases viral RNA expression in cis. When the METLL3 gene of host cells was knocked out or treated with 3DAA (m6A modification inhibitor) to inhibit m6A modification, the expression levels of viral mRNA and protein were reduced, which inhibited viral replication, while over-expression of YTHDF2 promoted the replication of IAV, which increases mature virions. m6A methylation in HIV-1 has also received much attention. Studies have revealed that m6A modification and the resulting recruitment of YTHDF protein are the main positive regulators of HIV-1 mRNA expression, and the HIV-1 protein/RNA expression and viral replication are enhanced by recruiting cellular YTHDF m6A ‘reader’ proteins [43]. However, other studies have shown that YTHDF1/2/3 proteins inhibit HIV-1 infection by blocking viral reverse transcription and promoting viral RNA degradation, or by reducing viral gRNA and early reverse transcription products [27, 44]. Therefore, the specific role of m6A methylation in regulating HIV-1 replication still needs further study. RNA regulation plays an important role in HCV infection. A study performed m6A analysis in HCV-infected cells and demonstrated that the HCV RNA genome is modified by m6A [23]. In HCV-infected Huh7 cells, inhibiting the expression of METTL3 and METTL14 significantly increased the abundance of HCV NS5A protein. In contrast, HCV NS5A levels were reduced when the m6A demethylase FTO was depleted. Another study used Huh7.5 CD81 knockout cells to test the effect of m6A on viral RNA replication or viral particle production, and the results showed that the m6A mechanism regulates HCV particle production but not HCV translation or RNA replication [45]. Taken together, these studies revealed that m6A, as a conserved regulatory marker in the HCV genome, negatively regulates HCV infection.

This is the first bibliometric analysis to systematically analyze publications related to m6A methylation in viral infection during the period 2000–2022. Different from traditional systematic reviews, the bibliometric analysis comprehensively quantifies and discovers research hotspots and trends in the target field through mathematical techniques. The research results enrich the knowledge base of viral infection-related methylation, which may help researchers analyze the characteristics, contribution distribution and knowledge map of m6A methylation in the field of viral infection, so as to find potential research topics, and may encourage further practice in this field. Nevertheless, there were some limitations in this study. Firstly, we searched only representative Web of Science core collection databases, and only for the period 1 January 2000 to 1 December 2022; some more recent published articles were not included. The retrieved literature may not be complete due to limited databases and time. Secondly, the extraction of some isolated keywords by the software was incomplete, although two bibliometric analysis software were used simultaneously. In addition, articles containing incomplete items were excluded, or insufficient research itself may also affect the accuracy of the results. In addition, the singleness of the analysis method may limit the comprehensiveness of the conclusion. Future research can improve the comprehensiveness of the conclusion by expanding data sources, improving analysis methods, and considering multiple analysis indicators. This will help to better depict the development trend of future research.

## Conclusion

In conclusion, m6A methylation in viral infection is an increasingly important topic in articles, and more scholars are devoted to the research of RNA methylation-related viral infection. With this study, we characterize the output of m6A methylation studies in viral infection, providing historical perspective. In this realm, China contributes the most in this area, while the US ranks highest in terms of total link strength, suggesting that articles published in the US may be more influential. This underscores the crucial need for bolstering collaboration among nations and institutions. Research on METTL3, ALKBH5, hepatocellular carcinoma, plant viruses, cancer progression, and Type I interferonthe current focal point and anticipated trend for future investigations. Nevertheless, numerous consequential knowledge voids persist, necessitating further investigations. Primarily, the molecular mechanisms regulating m6A modifications in viral infections are complex and diverse, demanding an extensive corpus of research evidence to unravel these intricate pathways. Secondly, with the advent of novel technologies such as single-cell sequencing and spatial transcriptomics, future exploration of m6A modification roles in viral infections can delve deeper. Lastly, future endeavors should emphasize the clinical applications of m6A modifications, harnessing their potential as therapeutic targets, fostering innovative antiviral therapies. In conclusion, this study holds pivotal significance in guiding future exploration into the role of m6A methylation in viral infections, offering crucial directions and a foundational framework for forthcoming investigations.

### Supplementary Information

**Additional file 1**. **Table S1**: The top 20 keywords related to m6A in viral infection.

**Additional file 2**. **Table S2**: The authors and cited authors related to m6A methylation in viral infection.

**Additional file 3**. **Table S3**: The top 10 co-cited journals related to m6A methylation in viral infection.

## Data Availability

The data are obtained through the public database. If a reasonable request is made, the corresponding author will provide the techniques used in this research.

## Abbreviations

m6A: N6-methyladenosine
IFN-I: Type I interferon
METTL3: Methyltransferase-like protein 3
METTL14: Methyltransferase-like protein 14
FTO: The fat mass and obesity-associated protein
YTH: Members of the YT521-B homology family of proteins
WoSCC: The Web of science Core Collection
LLR: Log-likelihood tests
KSHV: Kaposi’s sarcoma-associated herpesvirus
HIV-1: Human immunodeficiency virus 1
IAV: Influenza A virus
HCV: Hepatitis C virus
ZIKA: Zika virus
SV40: Simian vacuolating virus 40
EV71: Enterovirus 71
HMPV: Human metapneumovirus
HBV: Hepatitis B virus
RSV: Rous sarcoma virus
