# Supplementary material for: A bibliometric analysis of m6A methylation in viral infection from 2000 to 2022
Source: Virol J. 2024 Jan 18;21:20. doi: 10.1186/s12985-024-02294-1 (PMC10797797; doi:10.1186/s12985-024-02294-1)
Supplement: Supplementary file 1 — Additional file 1. Table S1: The top 20 keywords related to m6A in viral infection. [file 12985_2024_2294_MOESM1_ESM.docx]

| Table S1. The top 20 keywords related to m6A in viral infection. | | | | |
| --- | --- | --- | --- | --- |
| NO. | Keywords | Count | Year | Centrality |
| 1 | nuclear RNA | 123 | 2012 | 0.05 |
| 2 | methylation | 90 | 2016 | 0.09 |
| 3 | gene | 86 | 2000 | 0.22 |
| 4 | messenger RNA | 85 | 2001 | 0.12 |
| 5 | viral replication | 60 | 2012 | 0.07 |
| 6 | translation | 55 | 2018 | 0.03 |
| 7 | protein | 41 | 2017 | 0.12 |
| 8 | reveal | 38 | 2018 | 0.01 |
| 9 | expression | 34 | 2001 | 0.08 |
| 10 | recognition | 34 | 2017 | 0.04 |
| 11 | rna modification | 33 | 2019 | 0.04 |
| 12 | identification | 30 | 2012 | 0.11 |
| 13 | binding | 30 | 2016 | 0.09 |
| 14 | messenger rna methylation | 29 | 2017 | 0.09 |
| 15 | RNA methylation | 28 | 2019 | 0.02 |
| 16 | m (6) a | 26 | 2017 | 0.06 |
| 17 | virus | 26 | 2017 | 0.04 |
| 18 | m (6) a RNA | 23 | 2016 | 0.11 |
| 19 | innate immunity | 22 | 2019 | 0.05 |
| 20 | structural basis | 22 | 2017 | 0.07 |
