# Supplementary material for: A bibliometric analysis of m6A methylation in viral infection from 2000 to 2022
Source: Virol J. 2024 Jan 18;21:20. doi: 10.1186/s12985-024-02294-1 (PMC10797797; doi:10.1186/s12985-024-02294-1)
Supplement: Supplementary file 2 — Additional file 2. Table S2: The authors and cited authors related to m6A methylation in viral infection. [file 12985_2024_2294_MOESM2_ESM.docx]

| Table S2. The authors and cited authors related to m6A methylation in viral infection. | | | | | |
| --- | --- | --- | --- | --- | --- |
| Author | Count | Centrality | Cited author | Citation | Centrality |
| WANG X | 17 | 0.00 | WANG X | 181 | 0.01 |
| ZHANG Y | 16 | 0.03 | MEYER KD | 172 | 0.01 |
| LI Y | 15 | 0.03 | DOMINISSINI D | 153 | 0.01 |
| ZHANG X | 14 | 0.01 | LICHINCHI G | 144 | 0.00 |
| WANG Y | 14 | 0.00 | ZHENG GQ | 138 | 0.01 |
| WANG J | 13 | 0.11 | GOKHALE NS | 135 | 0.00 |
| CHEN J | 13 | 0.04 | JIA GF | 126 | 0.01 |
| SIDDIQUI A | 12 | 0.00 | LIU JZ | 121 | 0.02 |
| LI J | 12 | 0.07 | SHI HL | 112 | 0.01 |
| LIU Y | 11 | 0.10 | KENNEDY EM | 112 | 0.00 |
| HE C | 11 | 0.05 | ROUNDTREE IA | 102 | 0.00 |
| KIM G | 11 | 0.00 | LIU N | 100 | 0.01 |
| ZHANG W | 10 | 0.03 | TIRUMURU N | 92 | 0.00 |
