# Supplementary material for: A bibliometric analysis of m6A methylation in viral infection from 2000 to 2022
Source: Virol J. 2024 Jan 18;21:20. doi: 10.1186/s12985-024-02294-1 (PMC10797797; doi:10.1186/s12985-024-02294-1)
Supplement: Supplementary file 3 — Additional file 3. Table S3: The top 10 co-cited journals related to m6A methylation in viral infection. [file 12985_2024_2294_MOESM3_ESM.docx]

| Table S3. The top 10 co-cited journals related to m6A methylation in viral infection. | | | | |
| --- | --- | --- | --- | --- |
| Cited journal | Citation | Centrality | IF (2022) | JCR |
| *Nature* | 270 | 0.02 | 69.504 | Q1 |
| *Cell* | 267 | 0.06 | 66.850 | Q1 |
| *Nucleic Acids Research* | 250 | 0.01 | 19.160 | Q1 |
| *Proceedings of the National Academy of Sciences of the USA* | 239 | 0.02 | 12.779 | Q1 |
| *Molecular Cell* | 225 | 0.00 | 19.328 | Q1 |
| *Journal of Virology* | 203 | 0.01 | 6.549 | Q2 |
| *Nature Communications* | 199 | 0.01 | 17.694 | Q1 |
| *SCIENCE* | 197 | 0.01 | 63.714 | Q1 |
| *Cell Research* | 195 | 0.01 | 46.297 | Q1 |
| *Journal of Biological Chemistry* | 185 | 0.05 | 5.486 | Q2 |
